# Unraveling the Root Proteome Changes and Its Relationship to Molecular Mechanism Underlying Salt Stress Response in Radish (*Raphanus sativus* L.)

**DOI:** 10.3389/fpls.2017.01192

**Published:** 2017-07-14

**Authors:** Xiaochuan Sun, Yan Wang, Liang Xu, Chao Li, Wei Zhang, Xiaobo Luo, Haiyan Jiang, Liwang Liu

**Affiliations:** ^1^National Key Laboratory of Crop Genetics and Germplasm Enhancement, College of Horticulture, Nanjing Agricultural University Nanjing, China; ^2^School of Life Science and Food Engineering, Huaiyin Institute of Technology Huai'an, China; ^3^Jiangsu Key Laboratory for Horticultural Crop Genetic Improvement Nanjing, China

**Keywords:** radish, salt stress, iTRAQ, proteomics, association analysis

## Abstract

To understand the molecular mechanism underlying salt stress response in radish, iTRAQ-based proteomic analysis was conducted to investigate the differences in protein species abundance under different salt treatments. In total, 851, 706, and 685 differential abundance protein species (DAPS) were identified between CK vs. Na100, CK vs. Na200, and Na100 vs. Na200, respectively. Functional annotation analysis revealed that salt stress elicited complex proteomic alterations in radish roots involved in carbohydrate and energy metabolism, protein metabolism, signal transduction, transcription regulation, stress and defense and transport. Additionally, the expression levels of nine genes encoding DAPS were further verified using RT-qPCR. The integrative analysis of transcriptomic and proteomic data in conjunction with miRNAs was further performed to strengthen the understanding of radish response to salinity. The genes responsible for signal transduction, ROS scavenging and transport activities as well as several key miRNAs including miR171, miR395, and miR398 played crucial roles in salt stress response in radish. Based on these findings, a schematic genetic regulatory network of salt stress response was proposed. This study provided valuable insights into the molecular mechanism underlying salt stress response in radish roots and would facilitate developing effective strategies toward genetically engineered salt-tolerant radish and other root vegetable crops.

## Introduction

Soil salinity/salinization is emerging as one of the typical problems that global crops confront at present, and more than one-fifth of total croplands suffer from salinization process (Flowers and Yeo, 1995). Under salt stress, almost all major processes like seed germination, vegetative growth, flowering and fruit set are unfavorably disturbed, ultimately resulting in significant economic yield reduction and quality loss. Exposure to salinity can trigger severe disorders and disturbances in plants, including osmotic stress, ion toxicity/imbalance, reactive oxygen species (ROS) production and other secondary damages. Plants have enacted strategies to survive under these salt-induced damages. For example, osmotic adjustment can be achieved through accumulation of compatible solutes including proline, polyols betaine and soluble sugars, as well as the abatement of ion concentration in the cytoplasm. The ROS-scavenging enzymes and antioxidants can protect cells from salinity-triggered oxidative damage in crops such as wheat (Mandhania et al., 2006) and potato (Aghaei et al., 2009). Besides, salt tolerance also correlates with the alterations in levels of the phytohormones like abscisic acid (ABA), gibberellic acid (GA), ethylene and brassinosteroid (BR) (Deinlein et al., 2014). Therefore, to reduce potential risks of salinity to crop yield and quality, it is of great importance to reveal the molecular mechanism underlying salt stress response in plants.

Salinity tolerance depends on significant alterations in gene expression, and genes incarnate their functions through their protein products. Given the presence of post-transcriptional, translational and/or post-translational regulations, however, the alterations of genes at mRNA level cannot necessarily embody the same changes of the corresponding proteins (Fernie and Stitt, 2012; Chu et al., 2015). This highlights the high importance of proteomics because proteins are the direct players in plant responses to stress conditions. Consequently, the investigation at protein level could facilitate providing more accurate information to unveil complex mechanism underlying salt stress response.

Proteomics is a cutting-edge approach for proteome studies and widely adopted in the investigation on plant resistance to environmental stresses. Recently, proteomics-based technologies had been broadly employed to identify proteins responsible for salinity tolerance in several crop species such as cotton (Li et al., 2015), cucumber (Fan et al., 2015) and wheat (Jiang et al., 2017), promoting the understanding of changes in cellular activities under salt stress at protein level. For years, two-dimensional gel electrophoresis (2-DE) was one of the widely-used quantitative proteomics methods among biological samples. Nonetheless, the defects of low rate of protein detection, low reproducibility and difficult isolation of hydrophobic proteins restricted the full potential of 2-DE in systematic analysis of proteomic changes (Van den Bergh and Arckens, 2005). Isobaric tags for relative and absolute quantification (iTRAQ) analysis, a gel-free protein quantitative approach containing isotope labeling, has developed into one of the primary proteomic tools. Owing to its technical advantages of high accuracy and sensitivity and more-accurate quantification, iTRAQ had been successfully used in the investigation of DAPS under various abiotic stress conditions including temperature stress (Liu et al., 2014, 2017; Zhang et al., 2017), aluminum stress (Wang et al., 2014) and salt stress (Fan et al., 2015; Li et al., 2015; Xu et al., 2015a; Chen et al., 2016; Jiang et al., 2017), as well as biotic stresses like tobacco mosaic virus (TMV) infection (Wang et al., 2016) and powdery mildew infection (Fu et al., 2016).

Radish (*Raphanus sativus* L.), an important edible root vegetable belonging to Brassicaceae, is classified as a moderately salt-sensitive crop (Grattan et al., 2006). Roots are known as the primary sites that sense soil salinity and thus respond rapidly. Recently, the genome sequences of radish were successfully published (Kitashiba et al., 2014), providing a basis to break the bottlenecks that have narrowed radish genetic study. So far, however, limited information on proteomic change profiling related to salt stress response was available in radish. In this study, iTRAQ analysis was firstly employed to comprehensively assess the proteome dynamic changes under different salt treatments (0, 100, and 200 mM NaCl for 48 h) in radish roots. The potential biological functions of some protein species with significantly-altered abundance were investigated with the aim to explore their roles in salt stress response. Furthermore, the proteomic data were integrated with our previous miRNA and DGE data to unveil the associated genetic regulatory networks of radish response to salt stress. These results provided novel insights into genetic mechanism underlying salt stress response and would facilitate the genetic improvement of salt-tolerant radish and other root vegetable crops.

## Materials and methods

### Plant materials and salt treatments

The variety of ‘NAU-YH’, a small genotype with a globular shape, red skin and white flesh, was used in this study. A pre-experiment was established to survey the changes of visible physiological status under different concentrations of NaCl treatments (0, 50, 100, 200 and 300 mM) with hydroponic method. Interestingly, no special obvious morphologic differences were observed among individuals exposed to 50 mM NaCl, while the plants were seriously hampered and grew abnormally under 300 mM NaCl. The results showed that, under hydroponic culture of two salt-stressed conditions (Na100 and Na 200), some mature leaves of radish individuals were rolling, chlorosis, and the root enlargement and shoot elongation were relatively restrained. The growth conditions and salt treatments of radish plants were performed based on the reported descriptions (Sun et al., 2016). Plants were collected after 48 h with three different treatments including an untreated control (CK) and two salt-stressed conditions (Na100 and Na 200). For each treatment, an equal amount of fresh taproot samples from three individual radish plants were collected and pooled, and immediately frozen in liquid nitrogen. Ultimately, all harvested samples were stored at −80°C for further use.

### Protein extraction and quantification

Protein extraction of radish roots was conducted using the phenol extraction/methanol-ammonium acetate precipitation approach (Faurobert et al., 2007) with some modifications. Approximately 0.8 g of frozen radish roots was finely powdered in liquid nitrogen with 10% PVPP. The powder was then homogenized in 15 mL 10% TCA/acetone for 2 h, followed by centrifugation at 12,000 × g for 15 min at 4°C. The precipitate was then suspended in 5 × volume of protein extraction buffer (0.8 M sucrose, 100 mM KCl, 50 mM EDTA pH 8.0, 1 mM PMSF, 50 mM Tris–HCl pH 8.5, 1% DTT) for 15 min at 4°C. Afterward, an equal volume of cold Tris-buffered phenol (pH 8.0) was added, and then the sample was vortexed thoroughly for 10 min at room temperature. The mixture was centrifuged at 12,000 × g for 10 min at 4°C. The phenol phase was recovered carefully to avoid contact with the interphase and back-extracted using an equal volume of protein extraction buffer. The mixture was vortexed for 5 min at room temperature, and centrifugation for phase separation was repeated. The finally-recovered phenol phase was poured into a new tube and precipitated overnight with ten volumes of 0.1 M ammonium acetate in methanol at −20°C. After centrifugation at 12,000 × g for 10 min at 4°C, the supernatant was thrown away and the protein pellet was rinsed twice with cold methanol and thrice with 0.1 M cooled ammonium acetate in methanol and finally with cold acetone. The pellet was vacuum-dried at −20°C until the remaining acetone was evaporated, solubilized in 500 μL 0.5 M triethylammonium bicarbonate (TEAB), sonicated for 15 min, and then centrifuged at 12,000 × g for 20 min at 4°C. The supernatant was then quantified using the Bradford assay with BSA as the standard.

### Protein digestion, iTRAQ labeling and SCX fractionation

Total proteins of 100 μg from each sample were digested with Trypsin Gold (Promega, Madison, WI, USA) at 30:1 mass ratio at 37°C for 16 h, and then dried by vacuum centrifugation and reconstituted in 0.5 M TEAB. Labeling was processed with 8-plex iTRAQ reagent according to the manufacturer's manual (Applied Biosystems, USA). In brief, one unit of iTRAQ reagent was thawed and reconstituted in 24 μL isopropanol. The control sample was labeled with 117 iTRAQ reagent, and samples treated with 100 and 200 mM NaCl were labeled with 119, 121, respectively. The labeling reactions were incubated at room temperature for 2 h. After that, the peptide mixtures were pooled and dried under vacuum. For peptide fractionation, strong cationic exchange (SCX) chromatography was carried out with a LC-20AB HPLC Pump system (Shimadzu, Kyoto, Japan) as previously described (Chu et al., 2015; Fan et al., 2015).

### LC-ESI-MS/MS analysis

Each fractionated sample was resuspended in buffer A (5% ACN, 0.1% FA) and centrifuged at 20,000 × g for 10 min. Then, 10 μL (5 μg) each was loaded onto a 2 cm C_18_ trap column (inner diameter 200 μm) and eluted on a 10 cm analytical C_18_ column (inner diameter 75 μm) using Shimadzu LC-20AD nanoHPLC. The samples were loaded at 8 μL·min^−1^ for 4 min, after which a linear gradient was run at 300 nL·min^−1^ starting from 2 to 35% buffer B (95% ACN, 1% FA) for 35 min, followed by ramping up to 60% for 5 min, and then followed by 2 min linear gradient to 80%, and maintained at 80% buffer B for 4 min, and lastly returned to 5% within 1 min. All the mass spectral data were obtained by tandem mass spectrometry (MS/MS) using a Triple TOF 5600 System (AB SCIEX, Concord, ON) (Liu et al., 2015).

### Data analysis and protein identification

Raw data files were transformed into MGF files using Proteome Discoverer 1.2 (Thermo Fisher Scientific, San Jose, CA, USA). Protein identification and quantitation were simultaneously implemented using Mascot software version 2.3.02 (Matrix Science, London, UK) by searching against the radish Mixed Genome Database consist of publicly available radish genome database (ftp://ftp.kazusa.or.jp/pub/radish/) and three radish transcriptome databases (NCBI SRA accession No. SRX707630, SRX316199, and SRX1671013). All parameters were set as follows: Type of search: MS/MS Ion search; Digestion enzyme: trypsin; Fragment Mass Tolerance: 0.1Da; Peptide Mass Tolerance: 0.05Da; Mass Values: monoisotopic; Max Missed Cleavages: 1; Gln->pyro-Glu (N-term Q), Oxidation (methionine) and iTRAQ 8-plex (Y) were set as variable modifications; Carbamidomethyl (cysteine), iTRAQ 8-plex (N-term) and iTRAQ 8-plex (K) were set as fixed modifications. The charge states of peptides were set to +2 and +3. To reduce the false identification of peptides, only peptides at the 95% confidence interval were used, and each confident protein species contained at least one unique peptide. Only those peptides including at least two unique spectra were adopted for protein quantitation. The quantitative protein ratios were then weighted and normalized by the median ratio in Mascot. Protein species changed by >1.2- or <0.833-fold between any two treatments along with *p*-value <0.05 were defined as DAPS.

### Functional analysis

The identified protein species were assigned to the NCBI non-redundant (Nr) protein database using Blast2GO program to obtain their functional annotation. Gene Ontology (GO) database (http://www.geneontology.org) and Cluster of Orthologous Groups (COG) database (http://www.ncbi.nlm.nih.gov/COG/) were adopted to categorize these identified protein species. A metabolic pathway analysis was undertaken based on the KEGG Pathway Database. Furthermore, the GO and metabolic pathway enrichment analysis of the DAPS were conducted based on the information from the GO and KEGG pathway databases, respectively. As a result, those GO terms or pathways with *p*-value <0.05 were regarded as significantly enriched, which indicated the main biological functions or pathways of DAPS involved in salt stress response.

### RNA extraction and RT-qPCR

Radish root samples for iTRAQ analysis were adopted for RNA preparation. Total RNA extraction and reverse-transcription quantitative PCR (RT-qPCR) analysis were carried out according to our previously described method (Sun et al., 2016). All the primer pairs used for RT-qPCR were designed using the Beacon Designer 7.0 and provided in Table S1. *ACT* was set as an internal control for normalization and the NaCl-free radish roots were used as a reference sample whose value of expression level was set to 1. The data were processed with the 2^−ΔΔ*C*_T_^ method as described by Livak and Schmittgen (2001). The statistical analysis was carried out with SAS Version 9.0 software (SAS Institute, Cary, North Carolina, USA) according to Duncan's multiple range test at the *p* < 0.05 level of significance.

## Results

### Primary data analysis and protein identification

**Figure 1:**
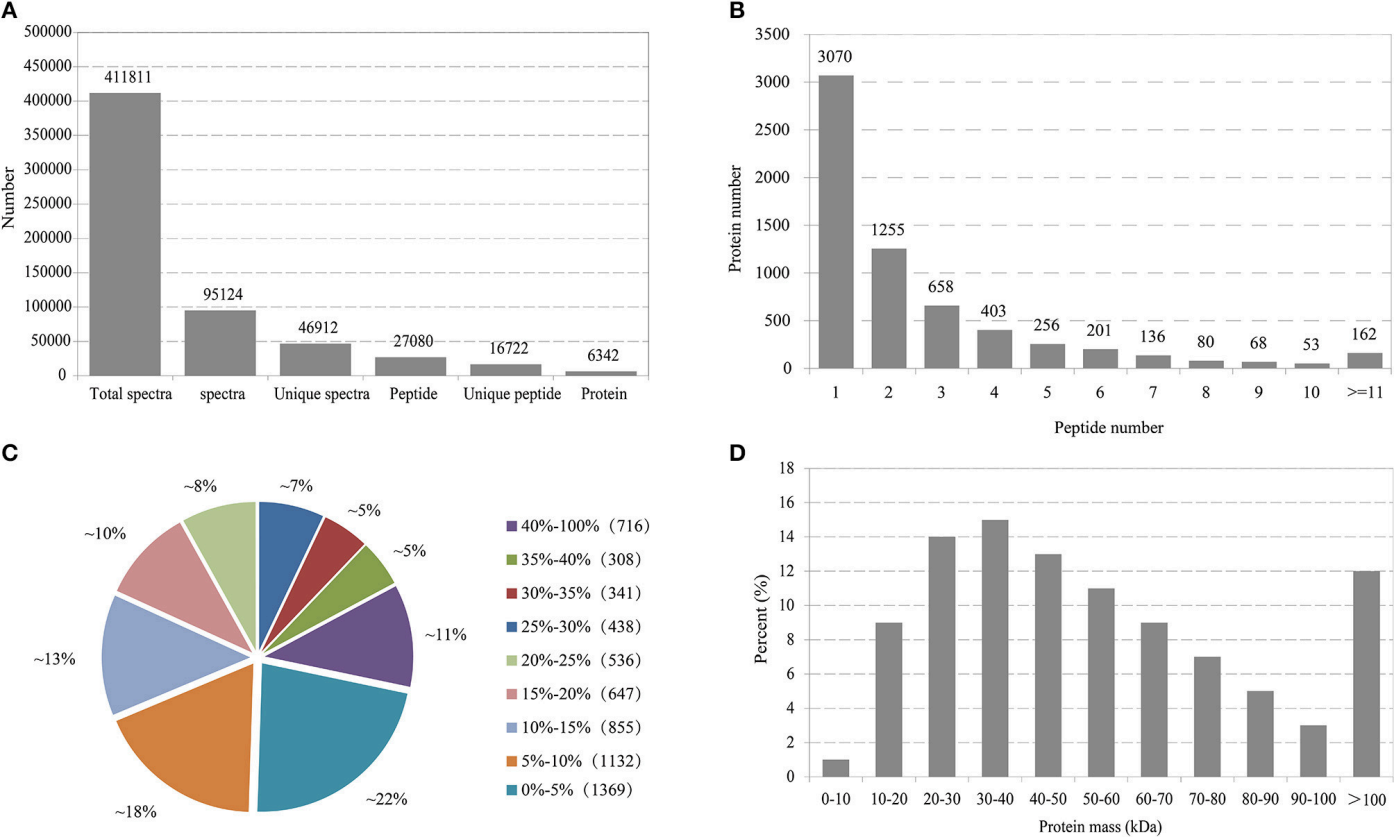
Primary data analysis and protein identification. **(A)** Basic information statistics. **(B)** Number of peptides that match proteins using MASCOT. **(C)** Distribution of protein's sequence coverage. **(D)** Protein mass distribution.

In total, 411,811 spectra were generated and 95,124 were matched to known spectra (Figure 1A). Among these identified spectra, 46,912 were unique spectra that were assigned to 27,080 peptides, with 16,722 unique ones. Finally, altogether 6,342 proteins were identified. The distribution of peptide number defining each protein was illustrated in Figure 1B and more than 51.5% of the unique proteins harbored at least two peptides. The coverage of over 60% of the peptide sequences was predominantly more than 10% and over 36% of the proteins constituted more than 20% of the sequence coverage, which suggested high confidence (Figure 1C). Regarding protein mass distribution, a good average coverage was achieved for ~86% of proteins from 10 to100 kDa (Figure 1D).

### Functional annotation and classification

**Figure 2:**
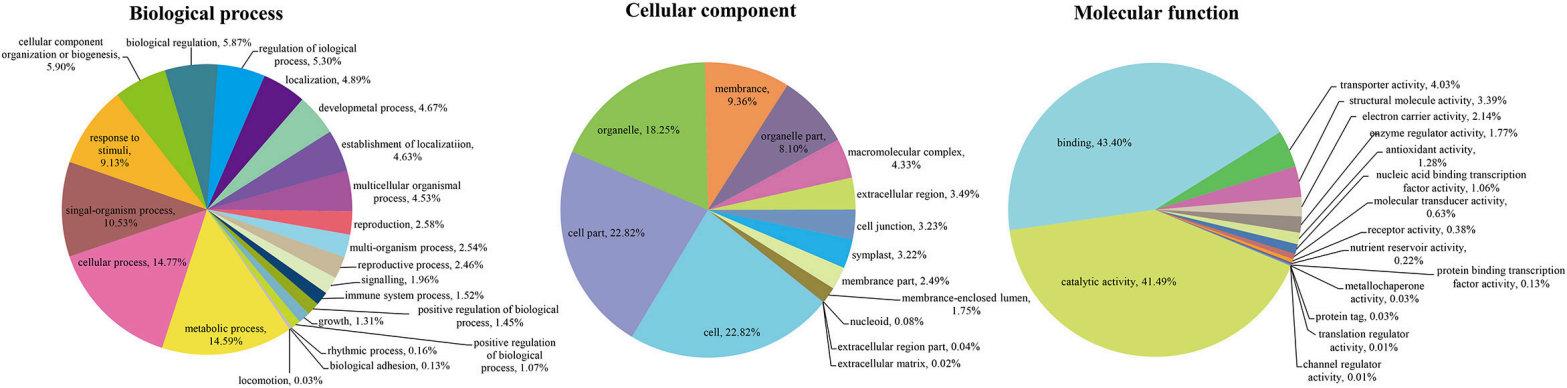
GO classification of the identified protein species.

To unveil the global analysis of protein species abundance, all identified protein species were searched against public databases including GO, COG, and KEGG to obtain their function annotation and classification. For GO analysis, these protein species were mainly classified into 52 important functional groups including 22 biological processes, 14 cellular components and 16 molecular functions (Figure 2; Table S2). According to the biological process properties, the most abundant terms were “cellular process” and “metabolic process” with 4,582 and 4,525 protein species, respectively. In cellular component category, “cell” (5,662 protein species), “cell part” (5662 protein species) and “organelle” (4,528 protein species) were the central categories. The “binding” and “catalytic activity” including 3,319 and 3,173 protein species, respectively, were the most dominant categories in molecular function.

**Figure 3:**
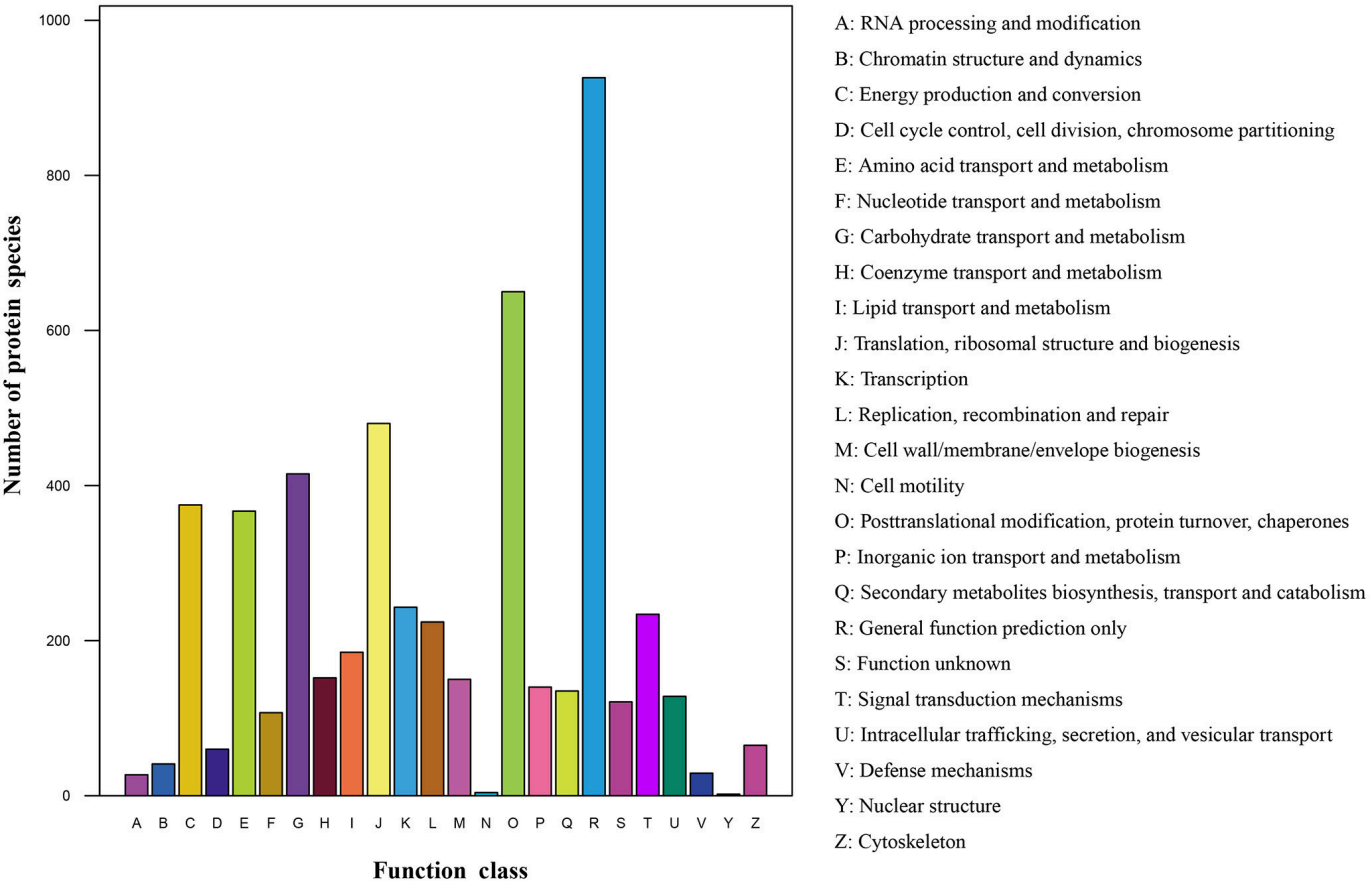
COG classification of the identified protein species.

All identified protein species with high homology were assigned to 24 COG categories (Figure 3; Table S3). The cluster for “general functions prediction only” (926 protein species) represented the largest functional category, followed by “Posttranslational modification, protein turnover, chaperones” (650 protein species) and “Translation, ribosomal structure and biogenesis” (480 protein species). However, only less than 10 protein species were assigned to “Nuclear structure” and “Cell motility” groups. Additionally, many protein species were clustered in several important categories related to stress responses such as “Signal transduction mechanisms” (234 protein species), “Transcription” (243 protein species), “Defense mechanisms” (29 protein species) and “Inorganic ion transport and metabolism” (140 protein species), indicating that these protein species play crucial roles in radish adaptive response to salt stress.

To characterize the primary metabolic pathways that protein species were implicated in, these protein species were further investigated using the KEGG database. In total, 128 KEGG pathways were successfully mapped for 4,560 protein species (Table S4), and the top ten pathways were shown in Table 1. The “metabolic pathways [ko01100]” (31.91%) and “biosynthesis of secondary metabolites [ko01110]” (17.98%) were the most dominant pathways.

**Table 1:** Top ten KEGG pathways for the identified protein species.

**Pathway**	**Protein species with pathway annotation**	**Pathway ID**
Metabolic pathways	1,455 (31.91%)	ko01100
Biosynthesis of secondary metabolites	820 (17.98%)	ko01110
Ribosome	222 (4.87%)	ko03010
Protein processing in endoplasmic reticulum	215 (4.71%)	ko04141
Spliceosome	171 (3.75%)	ko03040
RNA transport	169 (3.71%)	ko03013
Starch and sucrose metabolism	152 (3.33%)	ko00500
Plant-pathogen interaction	125 (2.74%)	ko04626
Phenylpropanoid biosynthesis	120 (2.63%)	ko00940
Purine metabolism	119 (2.61%)	ko00230

### Identification of DAPS

**Figure 4:**
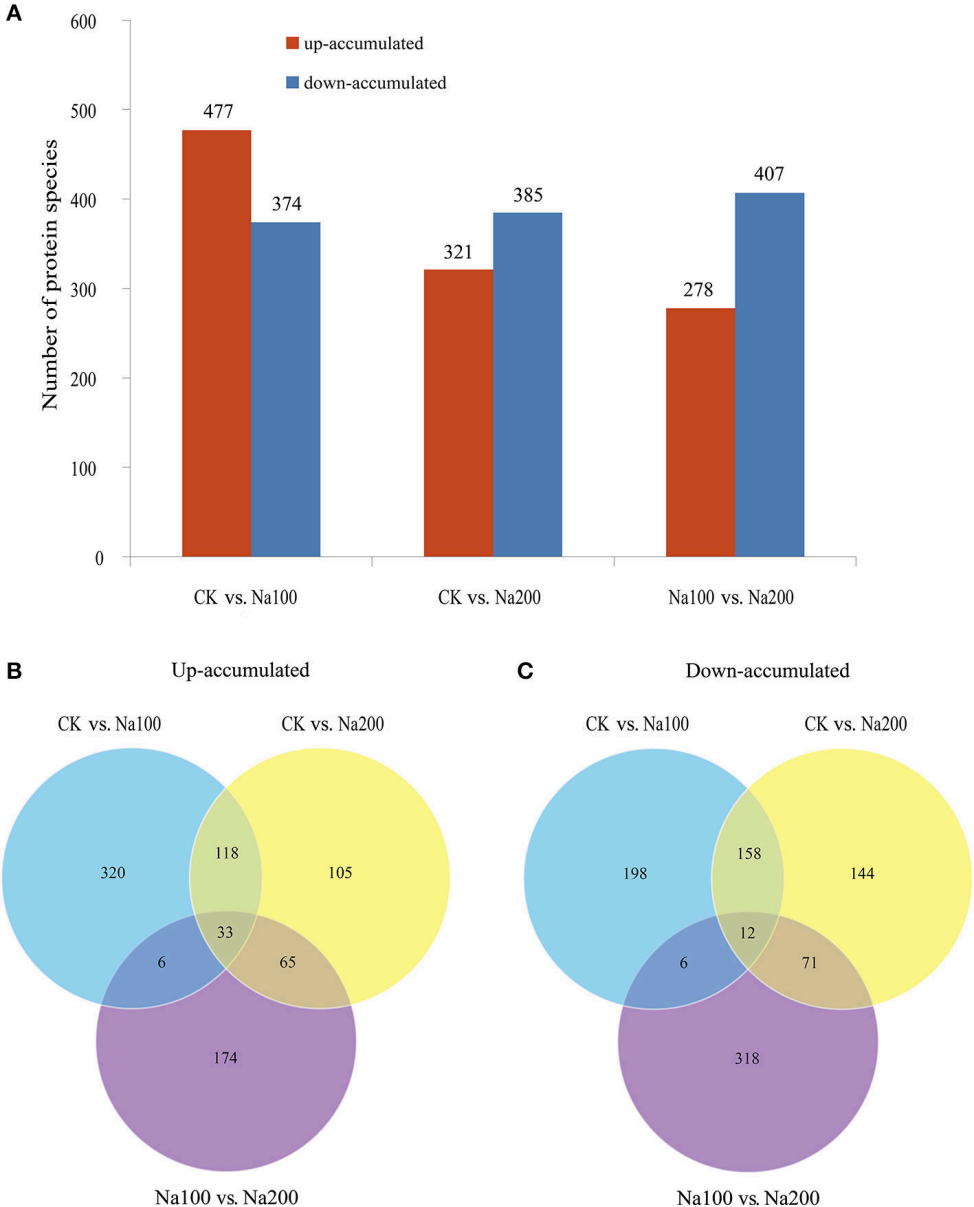
Identification and statistics of differential abundance protein species (DAPS) under salt stress. **(A)** Number of up- or down-accumulated protein species between any two different salt treatments. **(B)** Venn diagram analysis of up-accumulated protein species. **(C)** Venn diagram analysis of down-accumulated protein species.

**Figure 5:**
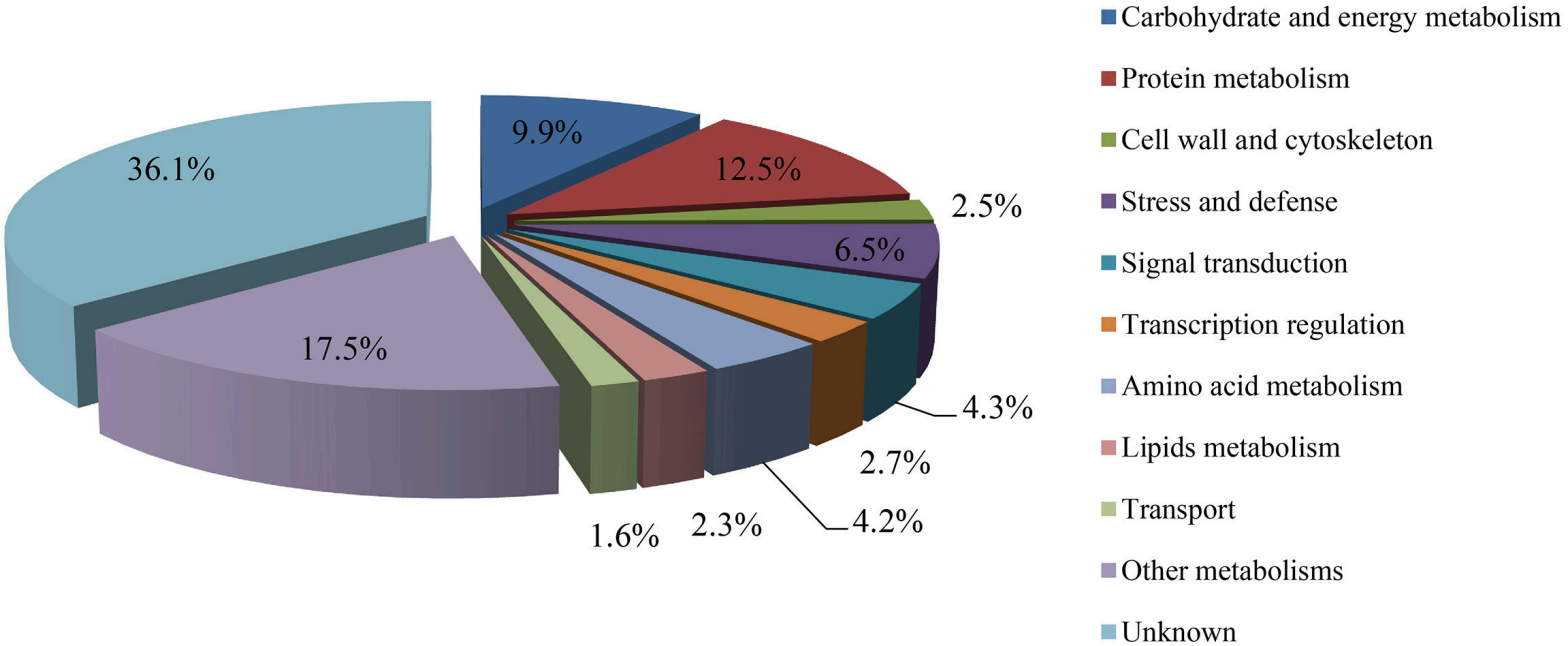
Classification of differential abundance protein species (DAPS) into cellular and molecular categories.

The protein species identified from radish roots between any two treatments (0, 100, and 200 mM NaCl) were quantitatively analyzed. Only these protein species whose levels changed more than 1.2-fold (up-accumulated) or less than 0.833-fold (down-accumulated) along with a *p*-value of less than 0.05 were considered as DAPS. Based on these criteria, 477, 321, and 278 protein species were identified to be up-accumulated, and 374, 385, and 407 protein species were down-accumulated between CK vs. Na100, CK vs. Na200, and Na100 vs. Na200, respectively (Figure 4A). This result showed that more protein species displayed altered abundance after 100 mM NaCl treatment. A Venn diagram for these up- and down-accumulated protein species between any two treatments was shown in Figures 4B,C. In addition, these DAPS could be further grouped into several cellular and molecular categories based on their potential functions in radish response to salt stress (Table 2; Figure 5; Table S5), including carbohydrate and energy metabolism (9.9%), protein metabolism (12.5%), signal transduction (4.3%), transcription regulation (2.7%), cell wall and cytoskeleton (2.5%), stress and defense (6.5%), transport (1.6%), amino acid metabolism (4.2%), lipid metabolism (2.3%), and other metabolisms (17.5%).

**Table 2:** List of differential abundance protein species (DAPS) identified in radish roots related to salt stress response.

**Accession**	**Protein species name**	**Organism**	**CK vs. Na100^a^**	**CK vs. Na200^b^**	**Na100 vs. Na200^c^**
**SIGNAL TRANSDUCTION**					
Rsa1.0_01545.1_g00007.1	14-3-3-like protein GF14 kappa	*A. thaliana*	1.474	0.955	0.642
Rsa1.0_00071.1_g00038.1	14-3-3-like protein GF14 kappa	*A. thaliana*	1.475	1.181	0.754
CL12477.Contig2_CKA	Annexin 1	*B. napus*	2.288	1.074	0.466
Rsa1.0_01707.1_g00009.1	Annexin E1	*B. oleracea* var. *capitata*	1.129	1.432	1.248
Rsa1.0_00094.1_g00025.1	Calcium-dependent protein kinase	*A. thaliana*	1.897	1.167	0.651
CL1474.Contig1_NAU-YH	Calcium-dependent protein kinase 2	*A. thaliana*	1.559	1.136	0.823
Rsa1.0_04961.1_g00001.1	Calcium-dependent protein kinase 21	*A. thaliana*	1.252	1.142	0.962
Unigene1570_CKA	Calmodulin	*Musa acuminata AAA Group*	1.677	0.779	0.493
Rsa1.0_00100.1_g00030.1	Calmodulin 5	*A. thaliana*	1.683	0.96	0.52
Rsa1.0_15163.1_g00002.1	Calmodulin-like protein 13	–	0.921	0.52	0.613
Rsa1.0_00517.1_g00005.1	Calmodulin-like protein 20	–	0.819	0.94	0.996
Rsa1.0_05431.1_g00001.1	COP9 signalosome complex subunit 4	*A. thaliana*	1.377	1.209	0.848
Unigene26735_CKA	Map kinase 3	*B. juncea*	1.601	2.056	1.275
CL7969.Contig2_NAU-LB	Nucleoside diphosphate kinase 1	*B. rapa*	1.441	1.332	0.921
Rsa1.0_05279.1_g00003.1	Phospholipase C	*A. lyrata* subsp. *lyrata*	1.352	1.306	0.958
CL2022.Contig4_NAU-YH	Phospholipase D	*B. napus*	1.552	1.314	0.857
Rsa1.0_00153.1_g00035.1	Ras-related protein RABA1b	*A. thaliana*	1.044	0.786	0.742
**TRANSCRIPTION**					
CL909.Contig2_NAU-YH	DNA-directed RNA polymerase II subunit RPB2	*A. thaliana*	1.316	1.014	0.726
Rsa1.0_05638.1_g00001.1	Histone H4	*Allium cepa*	2.421	0.747	0.376
CL61.Contig4_NAU-YH	Putative RNA helicase, DRH1	*A. thaliana*	0.597	0.783	1.301
Rsa1.0_05049.1_g00004.1	Splicing factor 3A subunit 2	*A. thaliana*	0.546	0.597	1.169
CL3347.Contig7_NAU-LB	Splicing factor like protein	*A. thaliana*	0.759	0.606	0.802
**PROTEIN METABOLISM**					
Rsa1.0_00370.1_g00008.1	17.6 kDa class I small heat shock protein	*A. lyrata* subsp. *lyrata*	0.473	0.347	0.902
CL1797.Contig1_NAU-LB	17.6 kDa class II heat shock protein	*A. lyrata* subsp. *lyrata*	0.37	0.48	1.034
CL3587.Contig3_CKA	20S proteasome subunit PAF1	*A. thaliana*	1.508	1.051	0.691
Rsa1.0_00123.1_g00059.1	26S protease regulatory subunit 8	*Oryza sativa Indica Group*	1.949	1.157	0.63
Unigene13987_CKA	26S proteasome non-ATPase regulatory subunit 10	*A. thaliana*	0.748	0.749	1.023
Rsa1.0_00006.1_g00049.1	26S proteasome subunit RPN6a	*A. thaliana*	3.512	2.65	0.748
Rsa1.0_00048.1_g00043.1	40S ribosomal protein S3A	*A. lyrata* subsp. *lyrata*	2.542	1.415	0.538
CL6296.Contig2_CKA	40S ribosomal protein S9	*A. lyrata* subsp. *lyrata*	1.985	1.161	0.612
Rsa1.0_00056.1_g00065.1	50S ribosomal protein L24-like protein	*A. thaliana*	1.476	1.3	0.943
Rsa1.0_00178.1_g00026.1	60S ribosomal protein L35	*A. lyrata* subsp. *lyrata*	1.824	0.821	0.471
Rsa1.0_00031.1_g00019.1	60S ribosomal protein L7A	*A. lyrata* subsp. *lyrata*	1.19	0.744	0.62
Rsa1.0_02836.1_g00003.1	Chaperonin 20	*A. thaliana*	0.574	0.613	1.161
Rsa1.0_00234.1_g00002.1	Chloroplast elongation factor tub	*A. lyrata* subsp. *lyrata*	0.839	0.744	0.908
Unigene26097_NAU-YH	Elongation factor 1B alpha-subunit 2	*A. lyrata* subsp. *lyrata*	2.273	2.89	1.239
Rsa1.0_00985.1_g00008.1	Eukaryotic initiation factor 4B	*A. thaliana*	0.877	1.361	1.455
Rsa1.0_00204.1_g00026.1	Eukaryotic translation initiation factor-5A	*B. napus*	0.745	0.771	1.038
Rsa1.0_00773.1_g00018.1	Peroxisomal small heat shock protein ACD31.2	*B. rapa*	0.638	1.229	1.911
Rsa1.0_00270.1_g00005.1	Polyubiquitin 3	*A. thaliana*	1.751	1.597	0.882
CL2440.Contig3_CKA	Protein disulfide isomerase	*B. carinata*	1.063	1.381	1.33
Rsa1.0_00695.1_g00005.1	Protein phosphatase 2c, putative	*Ricinus communis*	1.537	1.252	0.809
CL9610.Contig2_CKA	Putative protein disulphide isomerase	*B. napus* var. *napus*	3.348	1.161	0.304
CL73.Contig1_CKA	Translation initiation factor eIF(iso)4E.a	*B. rapa*	0.796	0.764	0.936
CL1668.Contig2_NAU-YH	Translation initiation factor eIF-3 subunit 3	*A. thaliana*	1.75	1.066	0.604
Rsa1.0_00121.1_g00017.1	Ubiquitin-like protein SMT3	*Cannabis sativa*	1.079	0.748	0.713
Rsa1.0_00153.1_g00039.1	Ubiquitin-conjugating enzyme E2 36	*Vitis vinifera*	0.709	0.767	1.089
CL1840.Contig1_CKA	Ubiquitin-conjugating enzyme E2 11	*A. thaliana*	0.735	1.093	1.476
Unigene21280_CKA	Putative ubiquitin-conjugating enzyme family	*Zea mays*	0.847	1.274	1.492
Rsa1.0_00329.1_g00001.1	Ubiquitin-associated /TS-N domain-containing protein	*A. lyrata* subsp. *Lyrata*	0.521	0.876	1.668
CL11046.Contig1_NAU-YH	Ubiquitin-like modifier-activating enzyme 5	*A. thaliana*	1.347	1.056	0.778
Rsa1.0_01220.1_g00012.1	Ubiquitin-conjugating enzyme 26	*A. lyrata* subsp. *lyrata*	0.69	0.732	1.181
**CARBOHYDRATE AND ENERGY METABOLISM**					
CL1927.Contig18_CKA	6-phosphofructokinase 3	*A. thaliana*	1.244	1.244	0.903
Rsa1.0_00451.1_g00021.1	Aconitate hydratase 1	*A. thaliana*	1.311	1.505	1.039
Rsa1.0_00732.1_g00001.1	Adenylate kinase	*A. thaliana*	1.066	1.293	1.256
CL6943.Contig1_CKA	Beta glucosidase 15	*A. thaliana*	1.279	0.768	0.719
CL1418.Contig1_NAU-LB	Beta-1,3-glucanase	*B. rapa* subsp. *chinensis*	1.141	12.169	10.629
CL4672.Contig3_CKA	Beta-amylase	*R. sativus*	3.119	1.695	0.614
Unigene2146_CKA	Enolase 1	*A. thaliana*	0.751	0.833	1.054
CL6906.Contig1_NAU-YH	Ferredoxin–NADP+ reductase-like protein	*A. thaliana*	0.406	0.651	1.564
Rsa1.0_42381.1_g00001.1	Fructokinase	*A. lyrata* subsp. *lyrata*	1.727	1.996	1.058
Rsa1.0_00490.1_g00017.1	Fructose-bisphosphate aldolase, class I	*A. thaliana*	0.24	0.304	1.258
Rsa1.0_04568.1_g00001.1	Fructose-bisphosphate aldolase, class I	*A. thaliana*	0.393	0.288	1.035
Rsa1.0_00891.1_g00009.1	Hexokinase	*B. oleracea*	1.551	1.005	0.603
CL7782.Contig1_CKA	Isocitrate dehydrogenase	*A. thaliana*	1.142	1.229	1.101
CL2656.Contig1_NAU-LB	Isocitrate dehydrogenase	*A. thaliana*	1.243	1.857	1.482
Rsa1.0_00168.1_g00014.1	Malate dehydrogenase	*A. thaliana*	0.477	0.758	1.59
CL9598.Contig2_CKA	NADH dehydrogenase	*A. thaliana*	1.586	0.989	0.623
CL13177.Contig1_NAU-YH	Phosphoenolpyruvate carboxylase	*B. juncea*	1.545	1.169	0.723
CL6916.Contig2_NAU-YH	Putative ATP synthase subunit	*A. thaliana*	1.41	1.968	1.383
CL4329.Contig1_CKA	pyruvate decarboxylase	*Phaseolus vulgaris*	1.813	1.906	0.995
Rsa1.0_00219.1_g00009.1	Pyruvate dehydrogenase E1 component subunit	*Cucumis sativus*	0.833	0.893	1.062
Rsa1.0_00801.1_g00012.1	Pyruvate kinase	*A. thaliana*	0.806	1.265	1.317
Rsa1.0_00231.1_g00009.1	Pyruvate kinase	*A. thaliana*	0.935	0.691	0.764
Rsa1.0_00176.1_g00013.1	Succinate dehydrogenase	*C. sativus*	0.999	0.696	0.711
Rsa1.0_15420.1_g00001.1	Sucrose synthase 1	*A. thaliana*	1.795	1.218	0.737
Rsa1.0_00204.1_g00019.1	UDP-glucose dehydrogenase 1	*A. thaliana*	0.576	0.749	1.403
**CELL WALL AND CYTOSKELETON**					
Rsa1.0_00622.1_g00009.1	Arabinogalactan protein 31	*A. thaliana*	1.322	0.816	0.617
Rsa1.0_06312.1_g00003.1	Chitinase class IV, partial	*B. napus*	1.228	4.317	3.489
Rsa1.0_01126.1_g00007.1	Class I chitinase	*Arabis blepharophylla*	1.235	0.585	0.476
CL4755.Contig1_NAU-LB	Expansin B1	*R. sativus*	0.831	0.681	0.913
Unigene23843_NAU-YH	Putative cytoskeletal protein	*A. thaliana*	1.772	1.411	0.796
Rsa1.0_00844.1_g00008.1	Putative polygalacturonase	*A. thaliana*	1.212	0.69	0.565
Rsa1.0_02233.1_g00003.1	Tubulin beta-2	*A. lyrata* subsp. *lyrata*	0.795	0.734	0.842
CL216.Contig1_NAU-YH	Xyloglucan:xyloglucosyl transferase	*A. thaliana*	2.09	1.624	0.664
**STRESS AND DEFENSE**					
CL3578.Contig2_NAU-LB	Adenosine kinase 2	*A. thaliana*	1.334	1.136	0.84
CL4092.Contig1_NAU-YH	Aldehyde dehydrogenase 2B7	*A. thaliana*	1.746	2.284	1.298
CL13563.Contig1_CKA	Ascorbate peroxidase	*B. oleracea* var. *italica*	1.144	1.469	1.573
Rsa1.0_11435.1_g00001.1	Catalase 3	*B. rapa*	1.672	0.909	0.622
Rsa1.0_01701.1_g00007.1	Superoxide dismutase	*R. sativus*	1.96	1.334	0.644
CL2774.Contig2_CKA	Dehydrin	*B. juncea*	1.219	1.326	1.055
CL2774.Contig1_CKA	Dehydrin protein	*Capsella bursa-pastoris*	1.263	0.873	0.73
CL2774.Contig2_CKA	Dehydrin	*B. juncea*	1.219	1.326	1.055
Rsa1.0_04198.1_g00002.1	Early-responsive to dehydration 4	*B. rapa*	1.551	1.193	0.771
CL7750.Contig1_NAU-LB	Glutaredoxin-C2	*A. thaliana*	3.366	5.792	1.707
Rsa1.0_13580.1_g00002.1	Glutathione peroxidase 2	*B. napus*	0.725	0.685	0.937
CL5893.Contig1_CKA	Glutathione reductase	*B. rapa* subsp. *campestris*	1.013	1.395	1.391
CL233.Contig5_CKA	Glutathione S-transferase 2	*B. juncea*	1.509	1.452	0.865
CL7659.Contig3_NAU-LB	Lipoxygenase 1	*B. napus*	1.924	1.47	0.824
Rsa1.0_00089.1_g00050.1	Lipoxygenase 2	*B. napus*	0.688	0.931	1.32
Rsa1.0_00089.1_g00053.1	Lipoxygenase 2	*B. napus*	1.513	0.818	0.56
Rsa1.0_02393.1_g00002.1	MLP-like protein 28	*A. thaliana*	0.777	1.602	1.979
Rsa1.0_00451.1_g00044.1	MLP-like protein 328	*A. thaliana*	0.586	0.459	0.828
Rsa1.0_02531.1_g00005.1	Peroxidase 13	*A. thaliana*	7.708	1.618	0.246
CL8877.Contig1_NAU-YH	Peroxidase	*A. lyrata* subsp. *lyrata*	1.016	0.435	0.439
CL14500.Contig1_CKA	Peroxidase	*Eutrema halophilum*	1.959	1.379	0.676
Rsa1.0_00230.1_g00008.1	Peroxidase 34	*A. thaliana*	1.781	2.216	1.132
CL4527.Contig1_NAU-YH	Thioredoxin m4	*A. thaliana*	0.665	0.683	1.091
**AMINO ACID METABOLISM**					
CL3871.Contig2_NAU-LB	Aspartate aminotransferase Asp2	*A. thaliana*	4.41	3.959	0.95
CL2420.Contig2_CKA	Chloroplast aspartate aminotransferase	*B. juncea*	2.867	4.049	0.917
Rsa1.0_02919.1_g00001.1	Glutamine synthetase	*R. sativus*	0.542	0.255	0.455
Rsa1.0_00143.1_g00011.1	Phenylalanine ammonia-lyase	*B. napus*	0.838	0.569	0.612
Rsa1.0_00087.1_g00123.1	Vitamin-b12 independent methionine synthase	*Populus trichocarpa*	1.348	1.004	0.704
**LIPIDS METABOLISM**					
CL11454.Contig1_CKA	Acetyl-CoA synthetase	*B. oleracea*	1.344	1.535	1.041
CL2282.Contig1_NAU-YH	Isovaleryl-CoA-dehydrogenase	*A. thaliana*	1.564	2.448	1.696
Rsa1.0_02286.1_g00004.1	lipase SIL1	*B. rapa* subsp. *pekinensis*	0.997	4.348	3.922
Rsa1.0_01285.1_g00010.1	Non-specific phospholipase C3	*A. thaliana*	1.782	1.122	0.694
CL3052.Contig1_CKA	Short-chain acyl CoA oxidase	*A. thaliana*	1.098	1.5	1.356
**TRANSPORT**					
CL14647.Contig2_CKA	Intracellular chloride channel-like protein	*A. thaliana*	1.34	1.124	0.864
Rsa1.0_01134.1_g00016.1	Plasma membrane aquaporin (PAQ2)	*R. sativus*	1.569	1.839	1.115
Rsa1.0_00707.1_g00021.1	Plasma membrane aquaporin 2b	*R. sativus*	2.096	1.624	0.882
CL683.Contig1_CKA	Plasma membrane aquaporin 2c	*R. sativus*	1.552	2.177	1.392
CL410.Contig1_NAU-LB	Vacuolar-type H+-ATPase subunit B3 (VHA-B3)	*A. thaliana*	1.346	1.258	0.877
CL334.Contig4_NAU-LB	VHA-A3	*A. lyrata* subsp. *lyrata*	1.609	1.379	0.772
CL6549.Contig2_NAU-LB	Voltage-dependent anion-selective channel protein	*B. rapa*	0.786	1.061	1.259
CL1962.Contig1_NAU-LB	V-type proton ATPase subunit E1	*A. thaliana*	1.543	2.193	1.41
Unigene2809_CKA	V-type proton ATPase subunit G1	*A. thaliana*	1.694	1.306	0.795
**OTHER MECHANISMS**					
CL60.Contig1_NAU-YH	ACC oxidase	*B. oleracea*	1.298	4.061	3.141
CL6450.Contig1_NAU-LB	Chalcone synthase	*B. juncea*	1.153	0.572	0.519
CL12290.Contig1_CKA	Flavanone 3-hydroxylase	*R. sativus*	0.542	0.294	0.505
CL4723.Contig1_CKA	Glyoxalase I	*Arachis hypogaea*	0.538	0.738	1.372
Rsa1.0_00239.1_g00017.1	Jasmonate inducible protein	*B. napus*	0.818	0.645	0.83
Rsa1.0_00293.1_g00001.1	Myrosinase	*R. sativus*	3.701	3.401	0.621
CL2892.Contig1_NAU-YH	Sulphite reductase	*A. thaliana*	1.321	1.657	1.242
CL4325.Contig3_NAU-YH	Vegetative storage protein-like	*A. thaliana*	4.995	6.163	1.272

a *The abundance ratio of proteins in radish roots under 100 mM NaCl compared to control for 48 h*.

b *The abundance ratio of proteins in radish roots under 200 mM NaCl compared to control for 48 h*.

c *The abundance ratio of proteins in radish roots under 200 mM NaCl compared to 100 mM NaCl for 48 h*.

### GO and KEGG pathway enrichment analysis for DAPS

**Figure 6:**
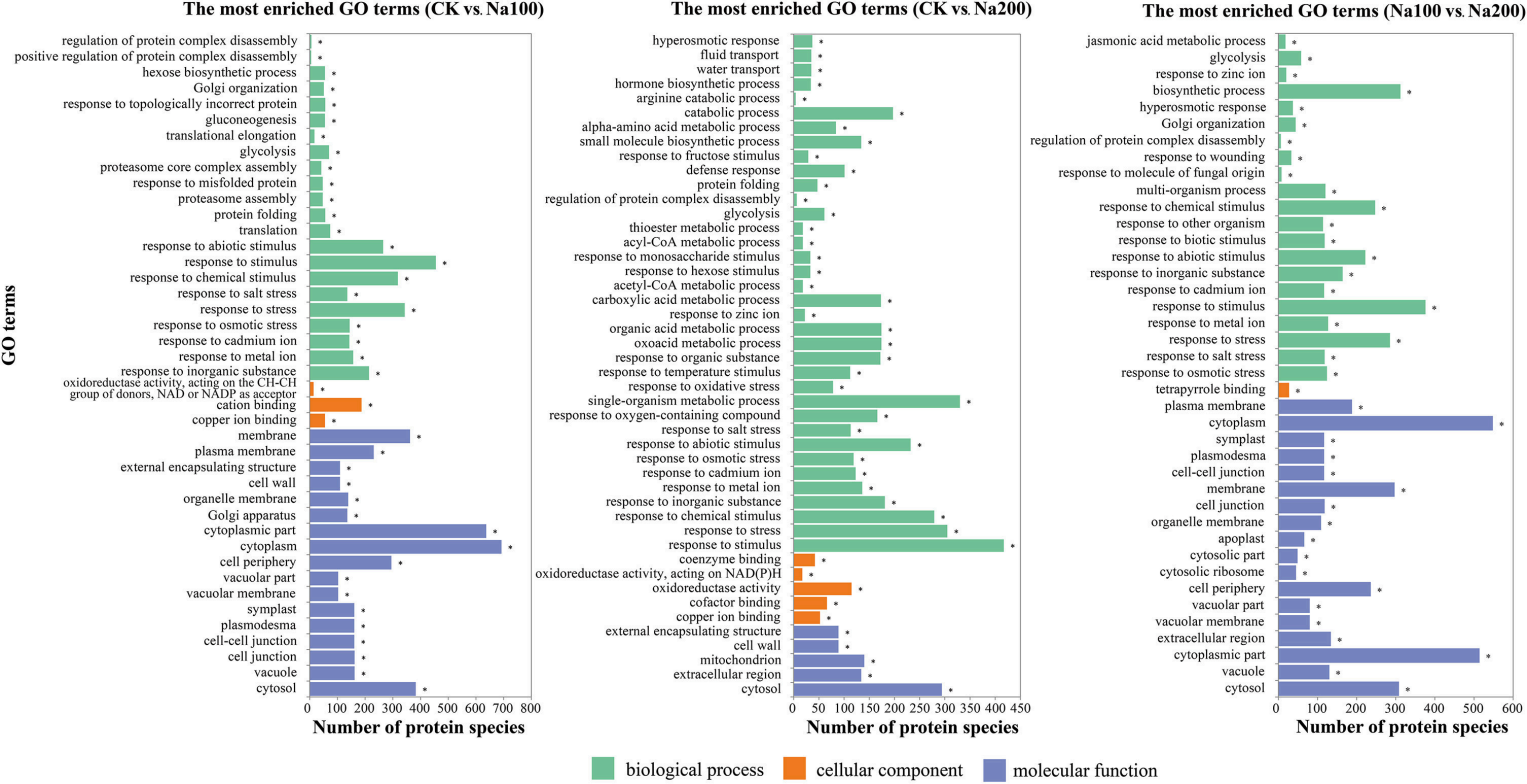
The most significantly-enriched GO terms of differential abundance protein species (DAPS) between any two different salt treatments (*p*-value ≤ 0.001).

On the basis of the GO enrichment analysis with a *p*-value < 0.05 as the threshold, a total of 289, 398, and 344 significantly-enriched GO terms representing three groups (biological process, cellular component and molecular function) were identified between CK vs. Na100, CK vs. Na200, and Na100 vs. Na200, respectively (Table S6), and the most enriched GO terms (*p*-value < 0.001) were shown in Figure 6. Among them, several significant cellular components were closely correlated with the transport activities of water and various ions including “vacuolar membrane” and “plasma membrane.” Numerous DAPS related to “calcium ion binding,” “calcium ion transport” and “calmodulin binding” were identified, potentially implying that calcium and calcium-mediated signaling pathway play important roles in the response to salt stress. Furthermore, many DAPS were found to be significantly enriched in stress response-related biological processes including “response to osmotic stress,” “hyperosmotic response,” “response to abiotic stimulus,” “response to salt stress,” and “response to oxidative stress,” which demonstrated that protein species involved in these biological processes were notably regulated to cope with various damages triggered by salt stress.

Pathway enrichment analysis exhibited that 11, 20, and 14 KEGG pathways were identified under salt stress between CK vs. Na100, CK vs. Na200, and Na100 vs. Na200, respectively, with a *p*-value ≤ 0.05 as the threshold (Table 3; Table S7). Of these, only 11 pathways were detected under 100 mM NaCl compared with untreated condition, such as “Citrate cycle (TCA cycle)” (2.71%), “Flavonoid biosynthesis” (2.33%) and “Glutathione metabolism” (3.1%). Moreover, only “Glycerolipid metabolism” (1.56%) and “Glycosphingolipid biosynthesis-globo series” (0.62%) were solely enriched between CK vs. Na100, and the enrichment of “Ether lipid metabolism” (0.98%), “Glucosinolate biosynthesis” (1.18%) and “Glycerophospholipid metabolism” (1.77%) was only detected between Na100 vs. Na200.

**Table 3:** Significantly enriched KEGG pathways for differential abundance protein species (DAPS).

**Pathway**	***p*****-value**			**Pathway ID**
	**CK vs. Na100^a^**	**CK vs. Na200^b^**	**Na100 vs. Na200^c^**	
Alanine, aspartate and glutamate metabolism	–	0.03786777	–	ko00250
Alpha-Linolenic acid metabolism	0.04201873	–	0.03604266	ko00592
Arginine and proline metabolism	–	0.002936132	–	ko00330
Beta-Alanine metabolism	–	0.006461845	–	ko00410
Biosynthesis of secondary metabolites	0.02396887	4.89611E-06	4.27007E-05	ko01110
Citrate cycle (TCA cycle)	–	0.01801409	–	ko00020
Cyanoamino acid metabolism	0.01564079	–	0.002374593	ko00460
Cysteine and methionine metabolism	–	0.006437393	0.005531991	ko00270
Ether lipid metabolism	–	–	0.01010504	ko00565
Fatty acid metabolism	0.02320015	0.01621388	–	ko00071
Flavonoid biosynthesis	–	0.008305518	–	ko00941
Glucosinolate biosynthesis	–	–	0.01414725	ko00966
Glutathione metabolism	–	0.01389174	–	ko00480
Glycerolipid metabolism	0.01908293	–	–	ko00561
Glycerophospholipid metabolism	–	–	0.03324163	ko00564
Glycolysis/Gluconeogenesis	0.0404229	0.000614053	–	ko00010
Glycosphingolipid biosynthesis-globo series	0.01702866	–	–	ko00603
Isoquinoline alkaloid biosynthesis	–	0.006409138	–	ko00950
Metabolic pathways	–	0.002096506	0.000302923	ko01100
Oxidative phosphorylation	0.01038897	–	0.01745488	ko00190
Phenylalanine metabolism	0.006216158	0.000422841	3.70086E-05	ko00360
Phenylalanine, tyrosine and tryptophan biosynthesis	–	0.04775363	–	ko00400
Phenylpropanoid biosynthesis	0.007688296	0.04727228	0.000552129	ko00940
Propanoate metabolism	–	0.000209769	0.04050957	ko00640
Pyruvate metabolism	–	4.81755E-05	0.009431396	ko00620
Ribosome	0.002021518	–	0.01969548	ko03010
Tropane, piperidine and pyridine alkaloid biosynthesis	–	0.03250334	–	ko00960
Tryptophan metabolism	–	0.0417651	–	ko00380
Tyrosine metabolism	–	0.001395104	–	ko00350

^*^ The detailed information for these metabolic pathways could be seen in Table S7.

a *The p-value of enriched KEGG pathways for DAPS under 100 mM NaCl compared to control for 48 h*.

b *The p-value of enriched KEGG pathways for DAPS under 200 mM NaCl compared to control for 48 h*.

c *The p-value of enriched KEGG pathways for DAPS under 200 mM NaCl compared to 100 mM NaCl for 48 h*.

### Verification of iTRAQ data by RT-qPCR

**Figure 7:**
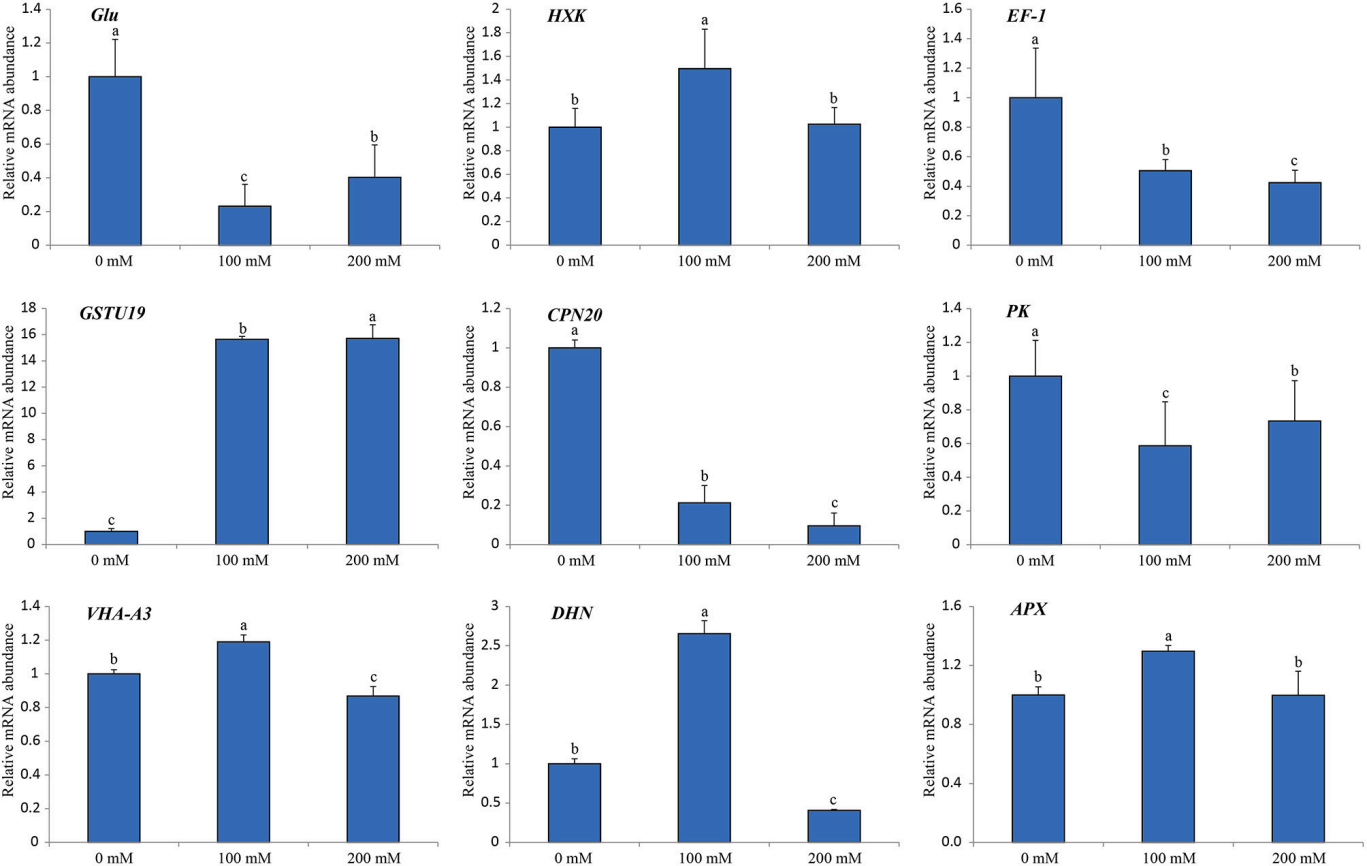
Relative mRNA expression analysis using RT-qPCR on nine protein species under diverse salt treatments. The expression level in the untreated samples (0 mM) was set to a value of 1. Each bar shows the mean ± SE (*n* = 3). Letters above the columns indicate significant differences at *p* < 0.05 according to Duncan's multiple range test.

In order to verify the iTRAQ data and investigate the correlation of protein species abundance with their corresponding mRNA level under salt stress, a total of nine protein species belonging to different cellular and molecular categories were randomly selected for RNA level examination using RT-qPCR (Figure 7). Of these, four genes (*HXK, EF-1, GSTU19*, and *DHN*) matched well in mRNA level with their translation products under different salt treatments. Moreover, our results also revealed that three genes encoding chaperonin 20 (*CPN20*; Rsa1.0_02836.1_g00003.1), VHA-A3 (CL334.Contig4_NAU-LB) and ascorbate peroxidase (*APX*; CL13563.Contig1_CKA) displayed similar protein and mRNA levels under different salt treatments. However, the gene encoding beta-1,3-glucanase (*Glu*; CL1418.Contig1_NAU-LB) showed decreased expression in response to salt, which was inverse with its protein abundance change. Apart from the different sensitivity between RT-qPCR and iTRAQ technique, a conceivable reason is that this poor correlation between mRNA and protein abundance may be arose from post-transcriptional, translational and post-translational regulations in the processes of mRNA translation and protein degradation.

### Integrative analysis on the changes of miRNAS, mRNAs, and proteins

In order to investigate whether the protein levels are correlated with the corresponding alterations in mRNA level, we firstly compared the proteomic data with our previous DGE data between CK vs. Na200 (Sun et al., 2016). According to the response of gene expression in protein level and mRNA level, the results could be classified into four groups: (1) both protein species and mRNAs showed a changed abundance (DAPS & DEGs; 102), including the same trends (56) and opposite direction (46); (2) protein species showed altered abundance without a change in mRNA level (DAPS & NDEGs; 308); (3) mRNAs expressed differentially without altered abundance in protein species (NDAPS & DEGs; 261), and (4) both mRNAs and proteins without altered levels (NDAPS & NDEGs; 1121) (Table S8; Figure S1). These results showed that, the correlation of genes in mRNA and protein level was relatively weak (*R* = 0.0354), indicating that an alteration in transcript level may or may not be translated into changes in protein abundance. Such a phenomenon might be due to the different regulatory mechanisms of genes in mRNA and protein level. Similar low correlation and limited correspondence between mRNA level and protein abundance were previously observed (Maier et al., 2009; Muers, 2011; Kubala et al., 2015).

Post-transcriptional regulation manipulated by miRNAs is a crucial regulatory mechanism for the modulation of gene expression, and ultimately affect protein abundance. To explore the impact of miRNAs on radish mRNA and protein abundance, we further performed an integrated analysis of miRNAs, mRNAs and proteins based on the previous miRNA data (Sun et al., 2015) and above mentioned DGE and proteome data. A total of 17 miRNA-mRNA pairs and corresponding protein species were identified (Table 4). Of these, almost all miRNAs and their corresponding mRNA targets had an anti-correlationship in expression, providing reliable evidence that miRNAs regulate functional gene expression in a negative manner. However, the abundance changes of target transcripts and correlated protein species only for four miRNAs (miR165a-3p, miR171a, miR408-5p, and miR414) were identified to be coincident, which was consistent with some results that the correlation between mRNAs and corresponding protein species was poor (Maier et al., 2009; Muers, 2011; Kubala et al., 2015).

**Table 4:** Association analysis of miRNAs, genes and protein species responsive to salt stress in radish.

**miRNA**	**LogFC^a^**	**Gene/transcript ID**	**LogFC^b^**	**Protein ID**	**LogFC^c^**	**Description**
miR156a	0.43	gi|332778243	−1.42	Rsa1.0_02678.1_g00001.1	–	Glutamine synthetase
miR156a	0.43	CL13383.Contig1_NAU-YH	−3.39	Rsa1.0_01634.1_g00012.1	0.10	Pentatricopeptide repeat-containing protein
miR156a	0.43	Unigene29157_NAU-YH	−1.26	Rsa1.0_00031.1_g00014.1	–	Transducin/WD40 domain-containing protein
miR157a	0.33	CL8020.Contig3_NAU-YH	−1.27	CL11389.Contig1_NAU-YH	–	Pentatricopeptide repeat-containing protein
miR157a	0.33	Unigene18175_NAU-YH	−1.62	Rsa1.0_00031.1_g00014.1	–	Transducin/WD40 domain-containing protein
miR165a	−1.44	gi|167430480	2.12	CL2529.Contig2_NAU-LB	−0.41	S-adenosylmethionine-dependent methyltransferase domain-containing protein
miR165a-3p	1.73	Unigene27908_NAU-YH	−0.98	Rsa1.0_00807.1_g00004.1	−0.34	Argininosuccinate synthase
miR171a	−2.40	CL8220.Contig1_NAU-YH	2.07	CL4933.Contig2_NAU-LB	0.04	Dehydrin ERD10
miR394a	1.72	gi|166138564	−9.57	CL3092.Contig1_CKA	0.45	Ankyrin repeat-containing protein
miR394a	1.72	CL4582.Contig2_NAU-YH	−9.92	Unigene2355_CKA	–	Mitochondrial substrate carrier family protein
miR394b-3p	1.06	Unigene18175_NAU-YH	−1.62	Rsa1.0_00031.1_g00014.1	–	Transducin/WD40 domain-containing protein
miR395a	−8.61	Unigene12970_NAU-YH	1.07	CL928.Contig2_CKA	–	ATP sulfurylase 1
miR396a	0.89	Unigene21235_NAU-YH	−1.47	Rsa1.0_01883.1_g00004.1	–	Glycosyl hydrolase family 38 protein
miR396a	0.89	Unigene26024_NAU-YH	−2.58	Rsa1.0_01809.1_g00003.1	–	RabGAP/TBC domain-containing protein
miR398b-3p	−1.94	CL4612.Contig2_NAU-YH	1.70	Unigene8101_NAU-LB	−0.01	Cu/Zn superoxide dismutase
miR408-5p	−1.17	gi|156153802	−5.16	Rsa1.0_00694.1_g00015.1	−0.13	Peptide chain release factor 1
miR414	−10.89	gi|161565681	4.49	Unigene26009_NAU-YH	0.09	Pentatricopeptide repeat-containing protein

*LogFC^a^Refers to the fold change of differentially expressed miRNAs in radish under 200 mM NaCl for 48 h compared to control (0 mM NaCl, CK), and the formula follows as: Fold change = log_2_(Na200/CK) (Sun et al., 2016)*.

*LogFC^b^Refers to the fold change of differentially expressed genes under same conditions (Sun et al., 2015)*.

*LogFC^c^Refers to the fold change of differentially expressed proteins under same conditions in this study*.

By exploring the potential functions of genes and their translation products, it was also found that several miRNA-mRNA pairs and corresponding protein species played a significant role in radish response to salt stress. For example, as the target of miR171a, dehydrin ERD10 was found to be accumulated in response to abiotic stresses, such as salt stress and drought, to protect cells against the consequences of dehydration (Kovacs et al., 2008). ATP sulfurylase 1 (*APS1*) was regulated by miR395a, which play a vital role in catalyzing inorganic sulfate assimilation (Matsui et al., 2013). Also, miR398-mediated Cu/Zn superoxide dismutase (*CSD1*) regulation was responsible for scavenging excess ROS generated in plants under salt stress (Jagadeeswaran et al., 2009).

## Discussion

Ultimate effects of plants upon perception of environmental cues are the proteome changes. Salt stress is one of the serious threats in agriculture. Progress in proteomics and advanced proteomics techniques like iTRAQ have made it possible to systematically detect the proteins and provide a global understanding of proteome changes during salt stress. In the current study, iTRAQ-based proteomics analysis was performed to investigate the molecular mechanism underlying salt stress response in radish.

### Calcium signaling in response to salt stress

In plants, calcium signaling pathway plays a crucial role in initiating complicated responses toward stress conditions. In our proteomic analysis, several key calcium-signaling components e.g., calmodulin (CAMs), calmodulin-like proteins (CMLs) and calcium-dependent protein kinases (CDPKs) were identified with altered abundance (Table 2; Table S5). It is well established that CAMs, CMLs, and CDPKs act as important Ca^2+^ signaling sensors in this signaling process (Srivastava et al., 2013). Furthermore, several other protein species related to calcium signaling pathway, including 14-3-3-like proteins, annexin, calreticulin, phospholipase C (PLC) and phospholipase D (PLD), were up-accumulated under salt stress (Table 2; Table S5). For example, 14-3-3 proteins can accomplish a key step in calcium-mediated signal transduction by binding to phosphorylated target proteins (Grant et al., 2015). Our results suggested that two 14-3-3-like protein species were significantly up-accumulated under salt stress especially 100 mM NaCl treatment, which was consistent with previous researches in salt-stressed sugar beet (Yang et al., 2012) and wheat (Wang et al., 2008). Generally, the abundance of the majority of these signaling-related protein species were strikingly increased by salt stress, implying that calcium signaling pathway plays an essential and positive role in radish response to high salinity.

### Salt stress induces the changes of protein metabolism

Protein modification, as well as the balance between synthesis and degradation, is a main regulation pathway which is coordinated to acquire a uniform cellular response to environmental stimuli (Hinkson and Elias, 2011). In eukaryotes, the ubiquitin/26S proteasome system is thought to be a repertoire of regulatory methods to selectively degrade the abnormal proteins and hence exert an essential housekeeping function (Vierstra, 2003). Moreover, the ubiquitin/26S proteasome system plays a vital role in almost all aspects of plant biology such as cell cycle, embryogenesis, signal transduction, circadian rhythms, senescence and stress tolerance (Vierstra, 2003). It is reported that the ubiquitin/26S proteasome system play critical roles in salt stress response (Fan et al., 2015; Li et al., 2015). In this study, 11 DAPS including three 26S proteasome and eight ubiquitin protein species were identified to be involved in the ubiquitin/26S proteasome pathway (Table 2; Table S5). Given that protein breakdown can not only supply molecular substrates for plant respiration but also activate plant responses to stress conditions (Araújo et al., 2011), thereby, it could be inferred that the ubiquitin/26S proteasome system has an essential potential to promote radish adaptation to salt stress.

In contrast to the ubiquitin/26S proteasome system, several types of protein species were related to protein biosynthesis and processing, such as ribosomal protein species, eukaryotic initiation factors (eIFs) and elongation factors (EFs). In translation, ribosome is the major site that the synthesis of a polypeptide chain takes place, and the ribosomal protein species play a role in translation, ribosomal structure and biogenesis. Our proteomic analysis showed that 40 ribosomal protein species mainly belonging to three types (40 S, 50 S, and 60 S) exhibited marked changes in abundance after salt treatments. A dominant number of these ribosomal protein species were elevated in abundance, potentially suggested that plants cope with salt stress by accelerating protein synthesis to maintain the balance between synthesis and degradation of proteins. Nonetheless, an obvious trend was observed that most of these ribosomal protein species (32/40, 80%) suffered a decrease in abundance from 100 to 200 mM NaCl treatment. A reasonable reason may be attributed to that the activity of ribosome is impaired with elevated intensity of salt stress. It is well known that eIFs and EFs play vital roles in translation initiation and elongation of peptide chain (Thornton et al., 2003). Strikingly, all five eIFs and five out of 10 EFs were down-accumulated by salt treatment. Generally, the differential regulation of distinct translation components suggested that protein biosynthesis might be managed by complex regulatory mechanism to cope with salt stress in radish.

### Salinity affects the protein species related to carbohydrate and energy metabolism

In plants, the glycolysis and tricarboxylic acid (TCA) cycle are principal features of carbohydrate and energy metabolism, which not only meet the energy demand but also give rise to many essential cofactors and substrates for other metabolisms (Plaxton and Podestá, 2006). In this study, 134 DAPS were correlated with carbohydrate and energy metabolism (Table 2; Table S5). Of these, the differential abundance of one hexokinase (Rsa1.0_00891.1_g00009.1), one 6-phosphofructokinase (CL1927.Contig18_CKA), two fructose bisphosphate adolases (Rsa1.0_00490.1_g00017.1 and Rsa1.0_04568.1_g00001.1), two pyruvate kinases (Rsa1.0_00801.1_g00012.1 and Rsa1.0_00231.1_g00009.1) and one enolase 1 (Unigene2146_CKA) suggested the presence of glycolysis required by plants as basic metabolism. Hexokinase is known as one of the key rate-limiting enzymes in the control of glycolysis by catalyzing glucose to glucose 6-phosphate. Fructose bisphosphate aldolase is a key glycolytic enzyme that has crucial potential in catalyzing an aldol cleavage of fructose-1,6-bisphosphate to dihydroxyacetone-phosphate and glyceraldehyde 3-phosphate in a reversible manner (Konishi et al., 2004). In the glycolytic pathway, a high-energy phosphoenol pyruvate is formed from 2-phosphoglycerate under the catalysis of enolase. However, the abundance of most of these identified glycolytic enzymes was notably decreased in our proteomic study, possibly indicating that the glycolysis activity was inhibited under salt stress in radish.

As downstream reaction for glycolysis products in mitochondria of aerobic organisms, TCA cycle is responsible for the oxidation of respiratory substrates to drive ATP synthesis assisted by various enzymes (Sweetlove et al., 2010). In the current proteomic study, the abundance changes of several key enzymes involved in TCA cycle including isocitrate dehydrogenase, pyruvate dehydrogenase E1 component subunit, succinate dehydrogenase and malate dehydrogenase were observed during salt stress. Isocitrate dehydrogenase is one of the most key three rate-limiting enzymes in TCA cycle. Two isocitrate dehydrogenases (CL7782.Contig1_CKA and CL2656.Contig1_NAU-LB) were up-accumulated, suggesting their positive potential in salt-stressed radish roots. However, the abundance of the remaining TCA cycle-related enzymes decreased with salt treatment. Especially, pyruvate dehydrogenase E1 is the component of pyruvate dehydrogenase complex, which exerts a key role in linking the glycolysis to the TCA cycle by catalyzing the formation of an acetyl-CoA from pyruvate (Vuoristo et al., 2015). This is a vital rate-limiting step reaction that determines the rate and efficiency of TCA cycle. Malate dehydrogenase is an oxidoreductase and reversibly catalyzes the interconversion of malate and oxaloacetate using NAD^+^ or NADP^+^ as coenzyme. Similar to glycolysis, these results also indicated that the TCA cycle activity suffered repression to some extent under salt stress, which is compatible with the results in salt-stressed cotton (Li et al., 2015) and heat-stressed grapevine (Liu et al., 2014).

### A common stress response induced by salt stress

The production of ROS, which irreversibly damage the cells and attack macromolecules, is one of the major stress-induced universal consequences in plants. Fortunately, the ROS can be scavenged by plant antioxidant defense system consist of a series of antioxidant compounds and enzymes. In the current study, the altered abundance of some important antioxidant enzymes, such as SOD, POD, CAT, Prx and GSTs, were observed during salt stress (Table 2; Table S5). SOD was increasingly accumulated in *Arabidopsis* (Jiang et al., 2007), wheat (Guo et al., 2012), and cotton (Li et al., 2015) during salt stress. In this study, the increment in the abundance of SOD (Rsa1.0_01701.1_g00007.1) suggested the significant involvement of this enzyme in sweeping salt-induced ROS in radish. PODs are capable of scavenging H_2_O_2_ using oxidation of co-substrates such as antioxidants (Meloni et al., 2003). Our results displayed that the abundance of six PODs were increased while two PODs were decreased by salt treatment. However, the abundance of three PODs (CL8877.Contig1_NAU-YH, CL14500.Contig1_CKA and Rsa1.0_02531.1_g00005.1) suffered marked decrease when salt concentration was increased to 200 mM NaCl, suggesting that the intensity of salt stress has a substantial influence on the complexity of stress response. Glutathione S-transferases (GSTs) are known as a family of well-characterized detoxification enzymes implicated in stress tolerance. Up-accumulation of GSTs in transgenic studies in rice (Takesawa et al., 2002) and tomato (Xu et al., 2015b) exhibited increased capability to harsh conditions such as low temperature, salt stress and drought. Our proteomic results showed that seven out of nine GSTs were found to be significantly up-accumulated by salt treatment, implying their significant roles in response to salt stress.

Lipoxygenases (LOXs) are emerging as a group of non-heme iron-containing dioxygenases catalyzing the degradation of polyunsaturated fatty acids to hydroperoxy fatty acids, which have detrimental effects on cellular membranes and proteins (Sofo et al., 2004). In this study, salt stress obviously enhanced the accumulation of LOX1 (CL7659.Contig3_NAU-LB) while two LOX2 (Rsa1.0_00089.1_g00050.1 and Rsa1.0_00089.1_g00053.1) displayed decreased abundance (Table 2; Table S5), suggesting that different LOX members may have differential functions in salt stress response in radish.

Additionally, some other protein species related to stress tolerance were also identified, such as dehydrins (DHNs) and universal stress proteins (USPs) (Table 2; Table S5). DHNs are known as a sub-family of late embryogenesis abundant protein species associated with desiccation resistance commonly accumulated in water deficiency or high salinity in plants. The abundance of two DHNs (CL2774.Contig1_CKA and CL2774.Contig2_CKA) was mildly enhanced under salt stress, suggesting that improved accumulation of DHNs potentially was an effective strategy to relieve salt-elicited water deficiency. USPs are broadly distributed proteins in nature. It is reported that USPs are helpful to constitute a natural biological defense mechanism to assist the organism in resisting unfavorable conditions (Loukehaich et al., 2012; Tkaczuk et al., 2013). However, in this study, all three USPs were significantly down-accumulated by salt stress, possibly implying that this type of proteins regulate the response of radish to salt stress in a distinct manner.

### Salinity affects the protein species involved in transport activity

The activity in influx, efflux, translocation and sequestration of solutes and multiple macromolecules across biological membranes is an essential part of cellular stress responses. Plasma membrane aquaporins are a class of channel proteins that facilitate the passive diffusion of water though cell membranes (Besserer et al., 2012) and play an important role in salt stress response. Katsuhara et al. (2003) reported that an aquaporin HvPIP2:1 from barley could raise salt sensitivity in transgenic rice under salt stress. The constitutive overexpression of a *GmPIP1;* six gene was reported to promote yield and salt tolerance in soybean (Zhou et al., 2014). Here, three plasma membrane aquaporins (Rsa1.0_01134.1_g00016.1, Rsa1.0_00707.1_g00021.1, and CL683.Contig1_CKA) were strikingly up-accumulated by salt treatment (Table 2; Table S5), suggesting that they acted as positive player in relieving salt-inducible damage in radish.

Many transport processes are dependent on the proton motive force that is achieved largely through the H^+^ gradient across membranes (Pittman, 2012). The vacuolar H^+^-ATPase (V-ATPase) is a multi-subunit enzyme complex that acts as a key enzyme for osmoregulation in numerous organisms by energizing secondary ion transport across the tonoplast (Dettmer et al., 2006). In the present study, the abundance of two V-ATPase subunits (VHA-A3 and VHA-B3) and two V-type proton ATPase subunits (E1/G1) were detected to be strikingly increased under saline condition (Table 2; Table S5), which agrees with the observations in *Arabidopsis* (Jiang et al., 2007), wheat (Guo et al., 2012), rice (Cheng et al., 2009) and tomato (Manaa et al., 2011), indicating their key roles in transport activities under salt stress condition.

### The regulatory network related to salt stress response in radish

**Figure 8:**
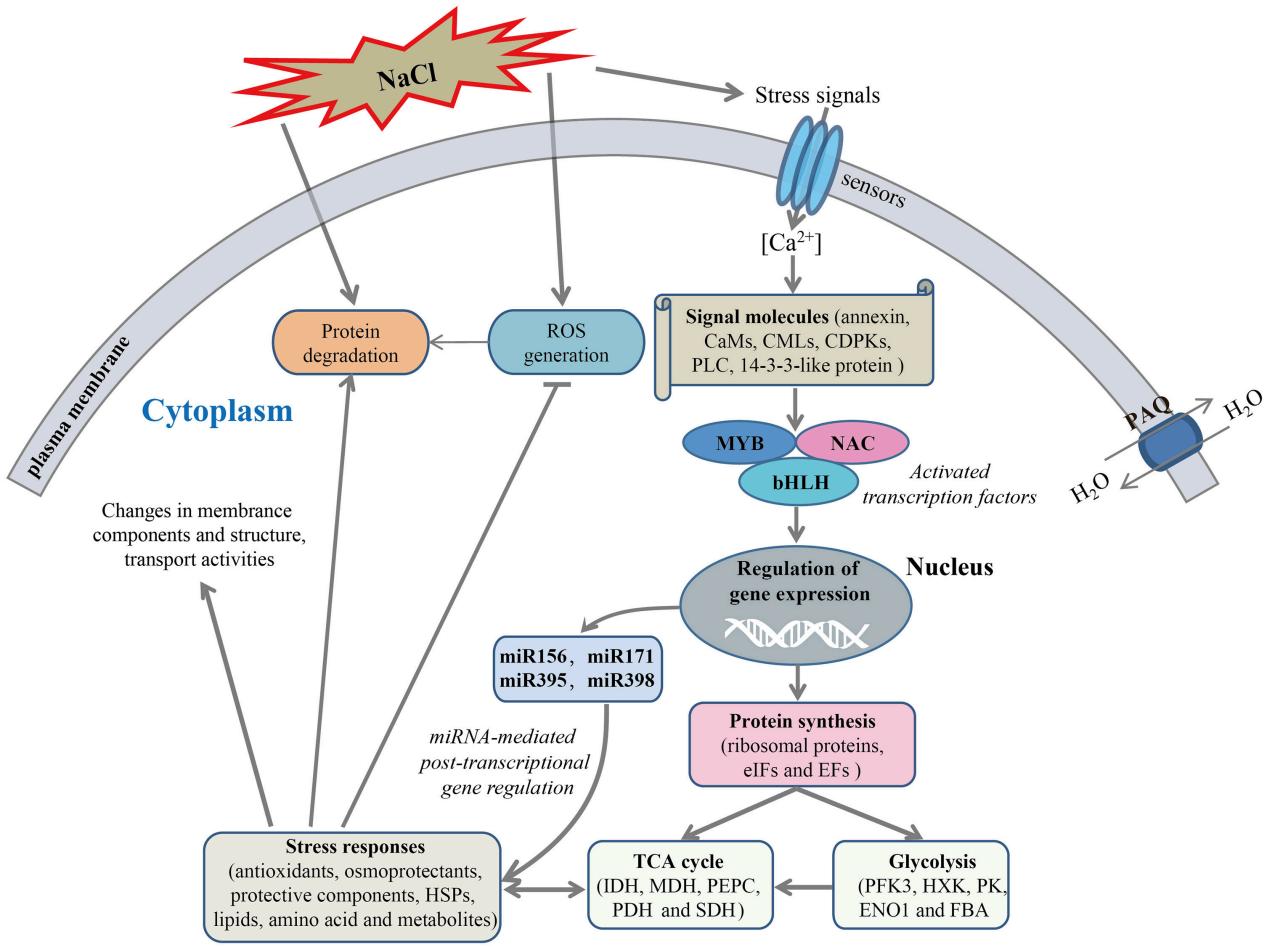
A schematic genetic regulatory network model of salt stress response in radish. The miRNAs and transcription factors (TFs) were identified in previous studies (Sun et al., 2015, 2016). ROS, Reactive oxygen species; CAM, calmodulin; CML, calmodulin-like protein; CDPK, calcium-dependent protein kinase; PLC, phospholipase C; MYB, myeloblastosis protein; NAC, (No Apical Meristem) domain-containing protein; bHLH, basic helix-loop-helix; PAQ, plasma membrane aquaporin; eIF, eukaryotic initiation factor; EF, elongation factor; PFK3, 6-phosphofructokinase 3; HXK, hexokinase; PK, pyruvate kinase; ENO1, enolase 1; FBA, fructose-bisphosphate aldolase; IDH, isocitrate dehydrogenase; MDH, malate dehydrogenase; PEPC, phosphoenolpyruvate carboxylase; PDH, pyruvate dehydrogenase; SDH, succinate dehydrogenase.

The mechanism of salt response is a very complex process that a variety of genes and response components involved in plants (Deinlein et al., 2014). In the present study, to reveal the molecular mechanism of salt stress response in radish, a schematic network model was put forward based on the abundant protein information obtained in this study in conjunction with association analysis of miRNA and transcriptomic data (Figure 8).

Once perceiving high salinity, the stress signals would induce the accumulation of Ca^2+^ in cytoplasm, which was essential for the activation of plant adaptive response to salt stress. With the aid of calcium-signaling molecules (e.g., PLC, CaMs, CMLs, CDPKs, and 14-3-3-like protein), the stress signals were transmitted and ultimately gave rise to the alterations in gene expression and protein abundance. Notably, transcription factors such as MYB, NAC, and bHLH were responsible for regulating the expression of downstream stress-responsive genes at the transcriptional level.

A direct consequence of high salinity was the disturbance in the balance of protein synthesis and degradation, which is essential to both cellular homeostasis and dynamics because almost all biological processes need the involvement of enzymes. Under salt stress, many key enzymes involved in the glycolysis and TCA cycle were severely affected, suggesting an inhibition of carbohydrate and energy metabolism. However, many cofactors and substrates generated in this process were essential for other metabolisms and stress response. Meanwhile, a series of defense responses were aroused to cope with salt-inducible injuries. For example, the antioxidant enzymes including SOD, POD, CAT, APX and GSTs were responsive for the scavenging of accumulated ROS caused by high salinity for the purpose of damage repair. The up-accumulated plasma membrane aquaporins could facilitate strengthening the water transport to alleviate dehydration of cells under salt stress. High salinity also regulated the abundance of some protein species enriched in cell wall metabolism, amino acid metabolism, lipid metabolism and other defense reactions, which is essential for salt tolerance. In addition, miRNA-mediated gene regulation at post-transcriptional level plays a vital role in plant response to salt stress (Sunkar et al., 2012). For instance, our study indicated that miR171a-targeted dehydrin *ERD10*, miR395a-targeted *APS1* and miR398-regulated *CSD1* were able to mitigate salt-induced dehydration (Kovacs et al., 2008), nutritional disorders (Matsui et al., 2013) and oxidative stress (Jagadeeswaran et al., 2009), respectively. Taken together, these findings provide a visualized insight into the molecular mechanism underlying salt stress response in radish.

## Conclusion

This large-scale proteomic study firstly provided a global view of proteome change under salt stress in radish roots. Totally, 851, 706, and 685 DAPS were identified during CK vs. Na100, CK vs. Na200, and Na100 vs. Na200, respectively. Functional annotation analysis for these protein species suggested that some DAPS related to several significant metabolisms, regulation of transcription, stress and defense and transport activities may play essential roles in enhancing radish tolerance to high salinity. In addition, the association analysis of miRNA, transcriptomic and proteomic data provided a strengthened understanding of radish response to salt stress at multiple levels. The genes associated with signal transduction, ROS scavenging and transport activities as well as several key miRNAs might be crucially responsible for salt stress tolerance in radish. Based on these findings, a schematic genetic regulatory network of radish response to salt stress was put forward. Overall, the outcome of this study will improve our understanding of salinity response mechanism and ultimately enable us to refine and improve yield and quality of radish and other important root vegetable crops for the benefit of humankind.

## Author contributions

XS, YW, and LL conceived and designed the research. XS, YW, and CL performed the experiments. WZ and XL contributed powerful analytical tools. XS and YW analyzed data. XS wrote the manuscript. LX and HJ revised the manuscript. All authors read and approved the final manuscript.

### Conflict of interest statement

The authors declare that the research was conducted in the absence of any commercial or financial relationships that could be construed as a potential conflict of interest.

## Supplementary material

Figure S1 Correlation for proteome and transcriptome between CK and Na200 treatments. Red spots represent both protein species and mRNAs showed a changed abundance (DAPS & DEGs); Blue spots indicate protein species showed altered abundance without a change in mRNA level (DAPS & NDEGs); Green spots show mRNAs expressed differentially without altered abundance in protein species (NDAPS & DEGs); and black spots represent both mRNAs and protein species without altered levels (NDAPS & NDEGs).

Table S1 Primers used for RT-qPCR analysis.

Table S2 GO term classification for the identified protein species.

Table S3 Functional classification of protein species by COG.

Table S4 KEGG pathways of the identified protein species.

Table S5 Differential abundance protein species (DAPS) under salt stress in radish.

Table S6 Significantly enriched GO terms for differential abundance protein species (DAPS).

Table S7 Significantly enriched KEGG pathways for differential abundance protein species (DAPS).

Table S8 Integrated transcriptomic and proteomic analysis of salt stress response in radish.

## Abbreviations

2-DE: Two-dimensional gel electrophoresis
iTRAQ: Isobaric tag for relative and absolute quantification
COG: Clusters of orthologous group
DAPS: Differential abundance protein species
DEG: Differentially expressed gene
RT-qPCR: Reverse-transcription quantitative PCR.
