# Supplementary figures and images for: Unraveling the Root Proteome Changes and Its Relationship to Molecular Mechanism Underlying Salt Stress Response in Radish (Raphanus sativus L.)
Source: Front Plant Sci. 2017 Jul 14;8:1192. doi: 10.3389/fpls.2017.01192 (PMC5509946; doi:10.3389/fpls.2017.01192)

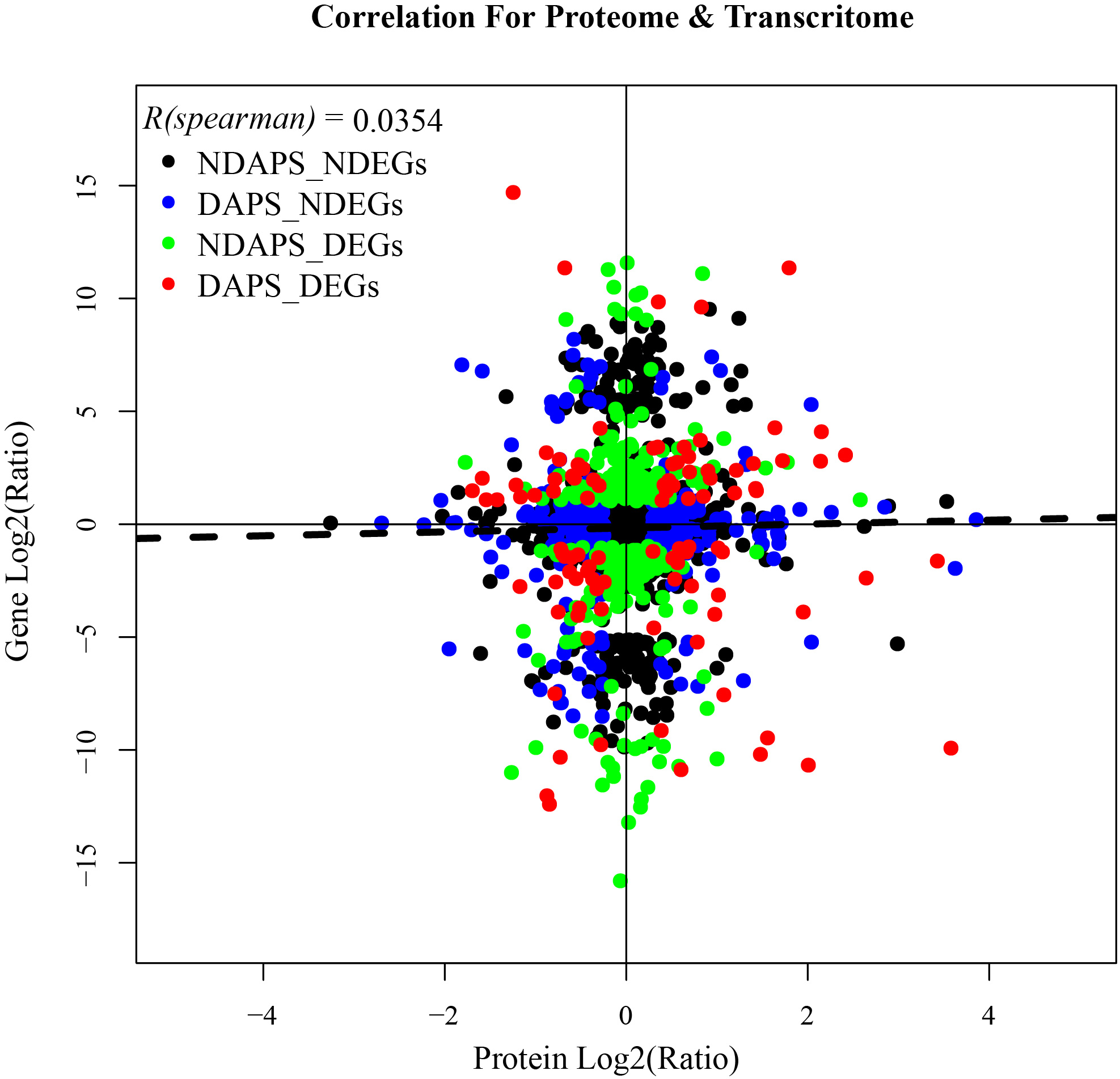

Supplement: Figure S1 — Correlation for proteome and transcriptome between CK and Na200 treatments. Red spots represent both protein species and mRNAs showed a changed abundance (DAPS & DEGs); Blue spots indicate protein species showed altered abundance without a change in mRNA level (DAPS & NDEGs); Green spots show mRNAs expressed differentially without altered abundance in protein species (NDAPS & DEGs); and black spots represent both mRNAs and protein species without altered levels (NDAPS & NDEGs). [file Image1.JPEG]
